# An unusual presentation of non pathological delayed splenic rupture: a case report

**DOI:** 10.4076/1757-1626-2-6450

**Published:** 2009-06-16

**Authors:** Suhail Aslam Khan, Izz Muhammad, Fadal Laabei, Jane Rothwell

**Affiliations:** 1 Department of Surgery, Naas General Hospital, Naas co, Kildare, Ireland; 2 Department of Surgery Adelaide & Meath Hospital, Tallaght, Dublin 24, Ireland

## Abstract

The diagnosis of Delayed Splenic Rupture poses a major challenge to even the most astute clinician, as it can mimic other medical emergencies. We present a case of an unusual presentation of delayed splenic rupture in a 23-year-old Caucasian man, who presented to the emergency department with a 2 day history of left upper quadrant pain. He initially denied any history of trauma. There were no signs of generalized peritonisim on examination but his haemoglobin level was low (8.9 gm/dl) for which there was no obvious cause identified. He was resuscitated and a computed tomography of the abdomen was performed. This revealed complete rupture of the splenic capsule with haemorrhagic fluid in the abdomen. With the computed tomography abdomen findings and further questioning of the patient, the only potential precipitating event that he could remember was a minor kick to the left upper quadrant more than 2 weeks ago while playing football. An urgent splenectomy was performed and histology confirmed complete rupture of the splenic capsule with a large adherent haematoma to the capsule. This case illustrates the difficulty in diagnosing delayed splenic rupture especially when accurate history is not available. A high index of suspicion is essential as delay in diagnosis can be fatal. Early diagnosis in suspected cases can be achieved by performing computed tomography of the abdomen.

## Case presentation

**Figure 1:**
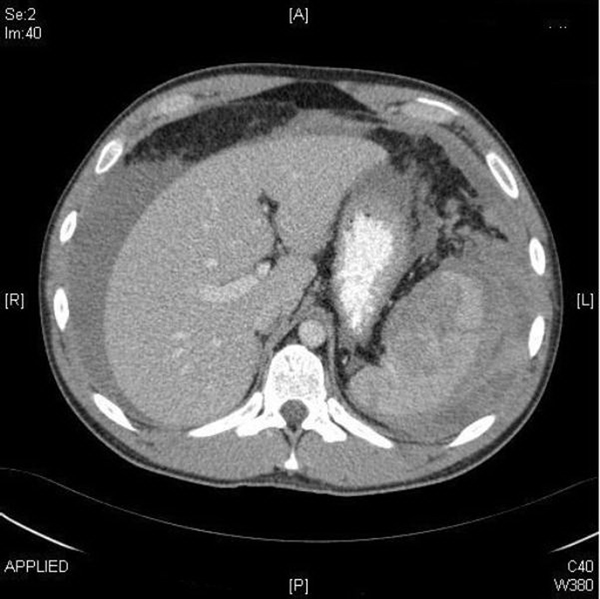
**Transverse section on CT Abdomen showing free fluid (blood) and laceration of spleen**.

**Figure 2:**
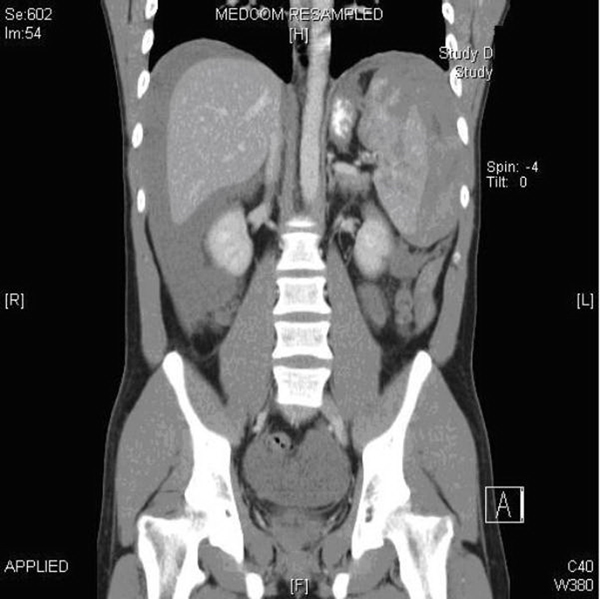
**Coronal section on CT Abdomen showing Haemoperitoneum and laceration of the spleen**.

**Figure 3:**
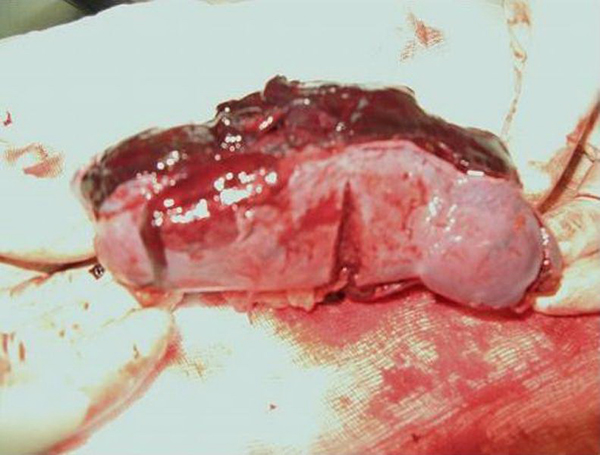
**Splenectomy specimen with evident sub-capsular haematoma**.

A 23-year-old Caucasian man attended the emergency department with a 2 day history of left upper quadrant pain and no other associated symptoms. He was referred by his GP who treated him initially with NSAIDs having little effect. He had no previous medical history and denied any trauma. He had no nausea, vomiting, diarrhoea, malaena or haematochezia. While he was in the emergency department, he experienced an episode of severe pain, which caused him to collapse. On physical examination, the patient was pale, anxious and in moderate distress. His blood pressure was 74/57 mmHg, pulse rate 82 beats per min and oxygen saturation 96% on 4 L of oxygen. His abdomen was tender to palpation and guarded on the left upper quadrant but there were no signs of generalized peritonism. Laboratory studies revealed a haemoglobin level of 8.9 g/dl, without any evidence of bleeding. After a bolus of 1L normal saline, his blood pressure increased to 106/85 mmHg. His initial radiographic studies, chest X-ray and plain film abdomen, were normal. After initial resuscitation, a CT abdomen (Figures 1,2) was arranged. This showed complete rupture of the splenic capsule with haemorrhagic fluid in the abdomen. Due to the CT findings, the patient was questioned again about any trauma in the recent past. The only potential precipitating event that he could remember was a minor kick to the left upper quadrant more than 2 weeks ago while playing football. He was completely asymptomatic during this time until presentation. Due to the findings of massive haemo-peritoneum found on CT scan of the abdomen, two units of packed red blood cells were transfused, and the patient was transferred to the operating theatre for emergency splenectomy. Intra-operative findings revealed a completely ruptured splenic capsule (Figure 3) with a large haemo-peritoneum. His post-operative recovery was uneventful, and he received the required vaccinations prior to discharge. Histological examination showed complete rupture of the splenic capsule with a large haematoma adherent to the capsule (sub-capsular haematoma) with no pathological abnormality of spleen.

## Discussion

The diagnosis of delayed rupture of the spleen may pose a major challenge to even the most astute clinician, as it can mimic other medical emergencies. Diagnosis maybe difficult due to the presumed triviality of the precipitating injury, an unpredictable time lag between the injury and the development of symptoms, and the possibility of atypical signs and symptoms remote from the bleeding spleen. The clinician may confuse the signs and symptoms with those of acute appendicitis or with some other cause. The authors present a case history to illustrate the diagnostic difficulties caused by delayed rupture of the spleen. Whenever the acute surgical abdomen is present with concomitant anaemia, the diagnosis of delayed rupture of the spleen should be considered.

Splenic rupture is usually reported in a pathologic spleen secondary to diseases such as infection, neoplasia and infiltrative processes. Delayed splenic rupture is not a new phenomenon and was described in the literature by Perasalo in 1949 [1]. The reason behind this case report is to emphasize the fact that the patient with blunt splenic trauma can present in many ways. The clinical signs and symptoms can vary widely where some patients being asymptomatic and others present in extremis.

Massive haemorrhage commonly occurs from injuries to this friable vascular organ. Usually diagnosis is easy in these cases. History of abdominal pain with left upper quadrant tenderness or signs of peritonitis in a patient is the most common presentation. Diagnosis is challenging where history is not relevant and clinical examination is not conclusive. The mortality rate from simple splenic rupture is 1 percent; delayed diagnosis of a ruptured spleen increases the rate to 10 percent [2]. Time is of the essence in these cases and early diagnosis is important to avoid grave morbidity or mortality.

The most common symptom is left upper quadrant pain and it is often associated with orthostatic symptoms [3]. Some unusual presentations have been reported with findings mimicking cardiovascular disease and scrotal hematoma [4].

Two signs are suggestive of splenic rupture: Kehr's sign (left diaphragmatic irritation resulting in referred pain to the left shoulder) and Balance's sign (palpable tender mass in the left upper quadrant [5]. Classically, patients can be found to be in hypovolemic shock with or without signs of peritonitis on abdominal examination. In fact, the diagnosis of splenic rupture is a diagnosis of exclusion and is not considered to be a primary diagnosis in the evaluation of patients with abdominal pain and hypotension. It is important to be aware of this rare clinical entity during practice because early recognition can be life saving.

Radiologic investigations play a major role in the diagnosis of delayed splenic rupture. Plain radiographs of the abdomen may show an elevated left hemi-diaphragm. In the emergency setting, CT scanning, which has supplanted angiography as the preferred diagnostic modality, clearly shows splenic hematoma or rupture. CT has a sensitivity and specificity of at least 95% in the detection of splenic injury [6]. Other investigations include visceral angiogram, focused abdominal sonographic technique (FAST) and diagnostic peritoneal lavage DPL method [7].

The treatment of delayed splenic rupture is debateable. There is no doubt that surgical intervention based on splenectomy is a necessity in patients with haemo-peritoneum and shock. The main controversy resides in the selection of patients who would benefit from a conservative approach. This is due to the fact that splenectomy patients require treatment in some form for their lifetime. Literature on this topic is available in the form of either case reports or personal experience but there is no clear cut data available. Both conservative and operative management is described but it is really subjective and depends on the presentation of patients symptoms.

For diagnosis and monitoring of patients being managed conservatively, contrast-enhanced computed tomography (CT) has been used. Stable patients with blunt splenic injury can be safely observed. However, there are certain risk factors in those requiring immediate surgery and those failing non-operative management. It must be bared in mind that CT scan underestimates injury, possibly related to a progression of bleeding found at the time of operation [8]. Other parameters used to monitor patients who are being managed conservatively is the degree of splenic injury by utilizing the Splenic Injury Scale as proposed by the American Association for the Surgery of Trauma (AAST), the presence of pseudo-cyst, or pseudo aneurysm. Another form of non-operative treatment would be use of splenic artery embolization treatment. It is observed that adequate use of splenic artery embolization for patients with blunt splenic injury avoids unnecessary surgery and avoids splenectomy [9].

Complaints of pain resulting from traumatic injury and abdominal examination findings did not identify patients requiring urgent operative management. Patients who are hemodynamic instable, evidence of multiple injuries, abnormal laboratory parameters, and the requirement for blood transfusion are likely to require operative therapy of their splenic injury [10].

Grading of splenic injury depends on the severity of radiological finding and by utilizing the Splenic Injury Scale by the American Association for the Surgery of Trauma (AAST), which extends from (Grade I) with only sub-capsular haematoma to shattered spleen (Grade V) injury. Analysis of the relation of the severity of organ injury to the use of non-operative management showed that degree I or II injuries could be treated non-operatively, whereas degree III and IV injuries could be treated with adhesives, partial resection, or mesh splenoraphy; while degree V injuries almost always required splenectomy [11]. Non-operative management of higher-grade splenic injuries is associated with a high rate of failure and prolonged hospital stay [12]. On the other hand, conservative management of low grade blunt splenic injuries had a 96% success rate if a strict protocol is followed [13].

In summary, the purpose of this report is to emphasize the importance of history and clinical examination in diagnosing the cause for the acute abdomen. In patients presenting with atypical symptoms and signs, history taking is of the utmost importance. Awareness of the problem of splenic rupture and an active diagnostic approach may help reduce the morbidity and mortality associated with splenic haemorrhage.

## List of abbreviations

CT: computed tomography; FAST: Focused abdominal sonographic technique; AAST: American Association for the Surgery of Trauma..

## Consent

Written informed consent was obtained from the parents of the patient for publication of this case report and accompanying images. A copy of the written consent is available for review by the Editor-in-Chief of this journal.

## Competing interests

The authors declare that they have no competing interests.

## Authors' contributions

SAK was the writer and surgeon. IM helped in proof editing and he is the assistant surgeon. FL was the consultant in charge of the patient. JR helped in proof editing and she was the supervisor.
